# Real-time Ultrasound-guided Lumbar Puncture: A Comparison of Two Techniques Using Simulation

**DOI:** 10.5811/westjem.21163

**Published:** 2025-05-20

**Authors:** Kara Samsel, David Wasiak, Elaine Situ-LaCasse, Srikar Adhikari, Josie Acuña

**Affiliations:** * Texas Tech University Health Sciences Center, Department of Emergency Medicine, El Paso, Texas; † The University of Arizona College of Medicine, Department of Emergency Medicine, Tucson, Arizona

## Abstract

**Introduction:**

The current literature on the use of real-time ultrasound-guidance for lumbar punctures (LP) is limited. Two techniques have been described: the paramedian sagittal oblique (PSO); and the transverse interlaminar (TL) approach. Our objectives in this study were to compare the procedure outcomes between these two techniques and assess the ability of emergency physicians to perform ultrasound-guided LPs.

**Methods:**

This was a prospective study whose participants included emergency physicians. Participants were randomized into either Group P (PSO technique) or Group T (TL technique). After a didactic session, participants then performed an ultrasound-guided LP on a training manikin, during which we collected procedure data. A survey was administered after completion of the procedure.

**Results:**

A total of 31 participants were included, 16 in Group P and 15 in Group T. Most participants (90.3%) successfully performed the procedure, without a statistical difference between Group P and Group T (15/16 vs 13/15, *P* = 0.95). Group T required a longer average time to complete the procedure (176.7 ± 140.4 seconds [s] vs 311.2 ± 202.3 s, *P* = 0.04). There was no statistically significant difference between Group P and Group T with regard to average time needed to obtain the required ultrasound view (18.3 ± 14.6 s vs 35.1 ± 32.9 s, *P* = 0.09); number of needle redirections; total number of needle passes; first puncture success; number of participants who advanced the needle without visualization of the tip (13/16 vs 14/15, *P* = 0.64); penetration of the anterior dura; and needle contact with bone. The Likert-style questionnaire responses (reported on a 1–10 scale) revealed no difference between Group P and Group T as to perceived difficulty of finding the required ultrasound view (3 [interquartile range (IQR) 2–5) vs 5 (IQR 3–6.5), *P* = 0.10), perceived difficulty of needle tracking, or rating of the needle view when entering the intrathecal space. However, Group T reported a higher overall perceived level of difficulty (4 [IQR 3–5] vs 6 (IQR 5.5–7.5), *P*= 0.01).

**Conclusion:**

This study suggests emergency physicians can be trained to use ultrasound-guidance for lumbar puncture in the simulation setting without significantly prohibitive training. Both techniques were performed with high success rates. There may be a preference for implementing the paramedian sagittal oblique approach over the transverse interlaminar.

## INTRODUCTION

Lumbar puncture (LP) is a valuable diagnostic and therapeutic procedure used in emergency medicine (EM) and other specialties for the evaluation and treatment of serious illnesses. Many procedures, including LP, have long been performed using “blind” landmark-based techniques, which depend on superficial anatomic structures as surrogate markers for deeper targets. The incorporation of ultrasound (US) has become more common with many of these procedures due to demonstrated increases in success rates and fewer complications.^1^,^2^ Procedural US may particularly benefit patients who are obese, have poorly palpable landmarks, or have atypical anatomy. It can be useful both for pre-procedure landmark identification (US-assisted) and real-time needle visualization during procedures (US-guided).

One of the most notable examples is central venous access.^3^ Ultrasound-guided central venous cannulation has garnered such positive support from the literature that it has been endorsed by the Agency for Healthcare Research and Quality, has been given a Level 1 recommendation by the American College of Emergency Physicians, and is now considered the standard of care.^4^,^5^ However, literature examining the use of real-time US-guidance for other procedures often used by emergency physicians, such as LPs, is less prevalent. Even more sparse is the literature evaluating the ability of emergency physicians to learn and perform the various techniques for US-guided LP.

Several US-guided LP techniques have been described in the literature. Two proposed methods are the paramedian sagittal oblique (PSO) approach and the transverse interlaminar (TL) approach.^6^–^12^ The two techniques differ in the ultrasonographic view of the interlaminar space that is used during the procedure. While the US-guided PSO and TL methods have been independently described in the literature, they have never been directly compared. In this study our goal was to compare these techniques using an adult LP simulator to assess for possible differences between the two techniques with regard to procedure outcomes (success rate, procedure time, and potential complications) and perceived difficulty of the procedure. Secondary objectives included assessing the effectiveness of a procedure training program.

## METHODS

This was a prospective study conducted at two academic medical centers with two categorical EM residencies and one combined EM/pediatrics residency program. There is an emergency ultrasound fellowship program affiliated with both centers and a robust training program for residents and faculty. This study received institutional review board approval.

Study participants included EM attendings and resident physicians with variable US and procedural experience. Participants were randomized into either Group P using the PSO technique or Group T using the TL technique. They completed a 30-minute training that included an instructional video and a hands-on session to practice identification of spine sonographic anatomy and real-time needle tracking. A low-frequency, curvilinear transducer was used for this study. Both methods use an in-plane technique for needle tracking in real time.

**Image 1:**
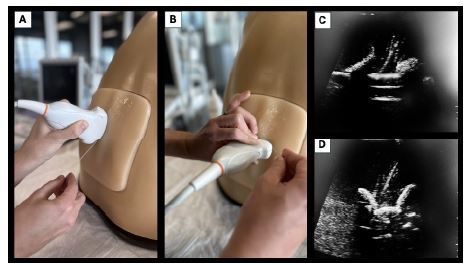
Representation of probe orientation and sonographic views for the paramedian sagittal oblique (PSO) and transverse intralaminar (TL) ultrasound (US)-guided lumbar puncture approaches. A. Photograph of the PSO approach to US-guided lumbar puncture. B. Photograph of the transverse intralaminar (TL) approach to US-guided lumbar puncture. C. US image depicting needle guidance using the PSO approach. D. US image depicting needle guidance using the TL approach.

To perform the PSO approach, the US probe is positioned in the sagittal direction, approximately 1 centimeter (cm) lateral to the midline above the sacrum and angled medially. The transducer is moved cranially until an interspace is reached and an optimal view into the spinal canal is obtained. Once a clear view is obtained, a puncture is performed paramedially, approximately 1 cm lateral to the midline on the opposite side. The needle is advanced into the field of the transducer. The target should directly lie beneath the ligamentum flavum in the spinal canal between the two adjacent laminae. For the TL view, the spinous process is first identified and centered on the US screen with the transducer in a transverse orientation. The transducer is then either moved cephalad or caudad to the interspinous space to identify the interlaminar space. The needle is introduced in a long-access approach with trajectory toward the interlaminar space, continuing until the intrathecal space is reached (Image 1).

**Population Health Research Capsule**

What do we already know about this issue?

*Two techniques for ultrasound-guided lumbar puncture (LP) include the paramedian sagittal oblique and transverse interlaminar approaches.*

What was the research question?

*We compared outcomes between the two techniques and assessed the ability of emergency physicians to perform simulated ultrasound-guided LPs.*

What was the major finding of the study?

*Most participants (90.3%) successfully performed the procedure, without a statistical difference between groups (P = 0.95).*

How does this improve population health?

*Emergency physicians can be trained to use ultrasound-guidance for LP in the simulation setting without time-intensive training.*

After the training session, participants performed an US-guided LP on a patient care manikin, during which we collected procedure data. As the study participants performed the LP procedure, an investigator observed the procedure and obtained the following information in real time: time to obtain the correct US view; total time to perform the procedure; success of procedure (ability to aspirate simulated cerebrospinal fluid); and number of skin punctures with the number of times the needle was redirected. Observers were emergency physicians, who were fellowship-trained in emergency ultrasound. After completion of the procedure, participants were surveyed on the perceived difficulty of the steps of the procedure, the helpfulness of the educational program, and the likelihood of considering the use of US-guided LP in the future. Participants were also asked to provide limited demographic information on the survey, such as level of training, prior experience with performing LP, and prior experience with performing US-guided procedures.

We summarized data by descriptive statistics using a confidence level of 95%. Procedure data were reported as the mean and standard deviation, while Likert-style questionnaire responses were reported as the median and interquartile range (IQR). We measured subjectively reported secondary endpoints obtained from the survey on a Likert scale. This included the perceived difficulty of the steps of the procedure, the helpfulness of the educational program, and the likelihood of considering the use of US-guided LP in the future.

## RESULTS

A total of 31 participants were included in this study, 16 in Group P and 15 in Group T. The characteristics of each group are outlined in Table 1. Of the 31 participants, 14 had prior experience using the US-assisted technique for LPs and two had experience using the US-guided technique. Procedural outcomes are presented in Table 2. The majority of participants (90.3%) successfully performed the procedure, without a statistical difference between Group P and Group T (15/16 vs 13/15, *P* = 0.95). Group T required a longer average time to complete the procedure (176.7 ± 140.4 seconds [s] vs 311.2 ± 202.3 s, *P* = 0.04). There was no statistically significant difference between Group P and Group T with regard to average time needed to obtain the required US view (18.3 ± 14.6 s vs. 35.1 ± 32.9 s, *P* = 0.09), number of needle redirections, total number of needle passes, first puncture success, number of participants who advanced the needle without visualization of the tip (13/16 vs 14/15, *P* = 0.64), penetration of the anterior dura, and needle contact with bone.

The post-questionnaire responses revealed no difference between Group P and Group T with regard to perceived difficulty of finding the required US view (3, [interquartile range] (IQR) 2–5) vs 5, IQR 3–6.5), *P* = 0.10), perceived difficulty of needle tracking, or rating of the needle view when entering the intrathecal space (Table 3). However, Group T reported a higher overall perceived level of difficulty (4, IQR 3–5) vs 6, IQR 5.5–7.5), *P* = 0.01). Additionally, survey respondents reported their likeliness of routinely using US-guidance for LP in the future (5, IQR 4–7), considering the use of US-guidance in difficult LP candidates (8, IQR 7–9), and considering the use of US-guidance in patients who have already had an unsuccessful LP attempt (9, IQR 7–10).

## DISCUSSION

Lumbar puncture remains a frequently used and important diagnostic procedure within the scope of EM. Although many procedures, such as central venous access, paracentesis, and thoracentesis have shifted toward the US guidance or assistance as the standard of care, LPs remain a procedure commonly done with only landmark guidance when performed in the emergency setting. Retrospective reviews and meta-analysis have shown that LPs using the standard landmark palpation technique have relatively high failure rates,^13^,^14^ whereas US-guided LPs are associated with higher success rates and fewer attempts.^15^–^17^ Ultrasound allows deeper structures and poorly identifiable landmarks to be visualized as opposed to identification through palpation alone. This may be of particular benefit to patients who are obese, have poorly palpable landmarks or atypical anatomy, and pediatric patients. The US-guided procedure provides the added benefit of using US to visualize the trajectory of a needle to its intended target in real time. Studies have shown that other procedures commonly performed in the emergency setting, such as central venous access and thoracentesis, have higher success rates and fewer complications when a real-time US-guided technique is used.^18^–^20^ However, the use of US-guidance for LPs in the emergency setting has been less explored.

The EM literature has long focused on the use of US-assistance to identify landmarks when performing LPs. It has been shown to decrease complications and the number of attempts when compared to the standard technique of palpating landmarks^21^,^22^ Literature, beyond case reports, on the use of US-guided LPs in EM has only emerged in recent years. When compared to the US-assisted technique, US-guidance has the benefit of improving safety by visualizing the needle during the procedure; however, this technique requires more advanced ultrasound skill, which could be seen as a deterrent for some emergency physicians. In addition to evaluating two US-guided techniques, we hoped to demonstrate that this was a skill emergency physicians could attain with training. The field of EM could benefit from larger prospective studies and, specifically, studies directly comparing guided vs assisted techniques when used by emergency physicians and their ability to translate training into the work environment.

Much of the existing knowledge on US-guided LPs is extrapolated from research in the field of anesthesia.^23^–^25^ Literature in this area focused on the use of US guidance to visualize and access the spinal canal for not only LPs, but for injections and nerve blockades. While many of these studies tend to use a PSO of the transducer, rather than TL, past reviews have found it difficult to come to any definite conclusion on whether this view is superior to others based on current existing literature.^26^ Of note, one prospective study performed in the perioperative setting evaluated the use of a paramedian transverse approach, similar to the TL approach used in our study. This study suggested that the paramedian transverse view may be superior as a means of achieving epidural access relative to a sagittal approach, since the paramedian transverse approach had significantly shorter procedure duration times and fewer number of attempts.^27^

The results of our study demonstrated that US-guided LP by both the PSO and TL had high success rates. Despite most of the participants not using US guidance for LPs previously, the instructional video and hands-on session provided enough training for the vast majority to succeed on the simulation models. Both the PSO and the TL approach provide an in-plane view for needle tracking throughout the lumbar puncture and had no difference in overall success. Although the views and approaches seem similar, some procedural differences were noted. For example, the PSO approach had a significantly decreased procedural time with an average of 176 seconds (s) vs 311 s on the TL approach (*P* = 0.04). The ease in obtaining the desired ultrasound view with a mean time of 18.3 was less in the PSO group relative to 35.1 in the TL group; however, did not meet statistical significance (*P* = 0.09).

The PSO group provided statistically fewer skin punctures as well without increasing the number of needle redirections and showing no significant difference in number of total needle passes. This information is of particular importance as many of the known complications of LPs (post-LP headache, infection, bleeding, radicular pain, paresthesias, back pain, spinal hematoma, and traumatic tap) are associated with the number of attempts. For example, one study found an association between multiple dural punctures and post-LP headache.^28^ Prior literature has found that multiple attempts and/or needle redirections are associated with more soft tissue damage, post-procedure pain, and traumatic taps.^29^–^31^ Additionally, we assessed whether the needle hit bone during the procedure, as this could lead to pain or bleeding from the innervated and vascular periosteum or penetrates the anterior thecal sac, which may cause injury to the anterior epidural venous plexus or intervertebral discs that lie just beyond the anterior dura. No significant differences in the occurrence of this was found between the two groups.

Findings from the post-procedural survey found no statistically significant difference in the overall ratings of difficulty for obtaining the ultrasound window for the PSO vs the TL method. This was also true for participants’ perceived difficulty of needle tracking. However, participants felt that overall, the PSO approach was less difficult than the TL approach. This was in agreement with some of the subjective findings obtained from procedural results. Subjective results found increased procedural time for the TL technique. There was also difficulty maintaining the view with needle in-plane as the majority of participants advanced the needle without the needle tip fully in view.

This study suggests emergency physicians can be trained to use US-guidance for LP. As mentioned, this study found that US-guided lumbar puncture by both the PSO and TL had high success rates. This was also in light of minimal instruction specific to these approaches prior to the study. There may be a preference for implementing the PSO approach over the TL view given its decreased overall procedural time and perceived level of difficulty.

## LIMITATIONS

This study has several limitations, among them the controlled environment of the simulation laboratory, which may not be reflective of an actual clinical scenario with a real patient. The study was performed on a standard LP manikin, which may limit generalizability given the difficulties in clinical practice including positioning and body habitus. For example, the depth from skin-to-spinous process and skin-to-intrathecal space is 3.5 cm and 5.5 cm, respectively. This depth will not translate to patients with large body habitus. Should this same study be performed on a model simulating large body habitus or perhaps spinal pathology (eg, scoliosis), we may have experienced different results. This underscores the need for future studies on live patients. An additional limitation is the small sample size, which may limit the study’s generalizability and the ability to identify small differences in performance. We also used a convenience sample of residents and attending physicians, which might have introduced selection bias.

## CONCLUSION

This study suggests emergency physicians can be trained to use ultrasound-guidance for lumbar puncture in the simulation setting without significantly prohibitive training. Both techniques were performed with high success rates. There may be a preference for implementing the paramedian sagittal oblique approach over the transverse interlaminar approach. Future studies should focus on the use of these techniques on live patients in the emergency setting.

## Figures and Tables

**Table 1:** Characteristics of emergency physicians who participated in simulation training for ultrasound**-**guided lumber punctures (LP).

Participant characteristics		Group P (n=16)	Group T (n=15)	P-value
Training level	PGY 1	0.250 (4/16)	0.333 (5/15)	0.91
	PGY 2	0.313 (5/16)	0.020 (3/15)	0.76
	PGY 3	0.125 (2/16)	0.067 (1/15)	1.00
	Attending	0.313 (5/16)	0.040 (6/15)	0.89
Number of ultrasounds performed	1–49	0.063 (1/16)	0.067 (1/15)	1.00
	50–149	0.063 (1/16)	0.200 (3/15)	0.95
	150–499	0.625 (10/16)	0.600 (9/15)	1.00
	≥ 500	0.250 (4/16)	0.133 (2/15)	0.71
Number of traditional LPs performed	1–14	0.625 (10/16)	0.533 (8/15)	0.88
	≥ 15	0.375 (6/16)	0.467 (7/15)	0.88
Number of in-plane procedures performed	0	0.063 (1/16)	0.200 (3/15)	0.55
	1–19	0.375 (6/16)	0.333 (5/15)	1.00
	≥ 20	0.563 (9/16)	0.467 (7/15)	0.86

*PGY*, postgraduate year.

**Table 2:** Lumbar puncture outcomes by group.

	Group P (n=16)	Group T (n=15)	P-value
Time to obtain ultrasound view (seconds)	18.33 (14.61)	35.13 (32.86)	0.085
Total procedure time (seconds)	176.69 (140.370)	311.20 (202.29)	0.043
Number of attempts (skin punctures)	1.81 (1.05)	3.13 (1.92)	0.042
Number of needle redirections	3.19 (3.31)	2.40 (2.72)	0.519
Total number of needle passes (attempts + redirections)	5.0 (3.85)	5.53 (3.27)	0.473
First puncture success	0.563 (9/16)	0.267 (4/15)	0.192
First pass success	0.188 (3/16)	0.133 (2/15)	1.00
Overall success	0.938 (15/16)	0.8 (13/15)	0.953
Hit bone with needle	0.313 (5/16)	0.533 (8/15)	0.378
Penetrated anterior dura with needle	0.5 (8/16)	0.4 (6/15)	0.843

**Table 3:** Participants’ post-procedural perceptions by group.

	Group P (n=16) Median [IQR] Mean	Group T (n=15) Median [IQR] Mean	P-value
Difficulty of obtaining ultrasound window	3 [2–5] 3.38	5 [3–6.5] 4.86	0.099
Difficulty of needle tracking	5 [4–6] 5.15	5 [4–7] 5.21	0.688
Rating of view of needle tip in spinal canal	7 [6–9] 7.08	7 [5.5–7] 6.79	0.335
Overall difficulty	4 [3–5] 4.38	6 [5.5–7.5] 6.36	0.008

*IQR*, interquartile range.
